# The interaction of Cu(II) and Zn(II) with peptide fragment of HSPB1 and its analogs

**DOI:** 10.3389/fmolb.2025.1593661

**Published:** 2025-09-04

**Authors:** Agnieszka Szebesczyk, Domenica Musumeci, Ettore Napolitano, Halyna Kukhtenko, Paulina Iwaniak

**Affiliations:** ^1^ Institute of Health Sciences, University of Opole, Opole, Poland; ^2^ Department of Chemical Sciences, University of Naples Federico II, Naples, Italy; ^3^ Department of Cosmetology and Aromology, National University of Pharmacy, Kharkiv, Ukraine; ^4^ Faculty of Medicine, Medical University of Lublin, Lublin, Poland

**Keywords:** copper, zinc, heat shock proteins, metal ions binding sites, peptides, ala-screening

## Abstract

**Introduction:**

Copper (II) and zinc (II) ions are essential microelements in the human body, interacting with numerous biologically active molecules, including proteins and peptides. The precise identification of binding sites, complete with the detailed characterization of binding amino acid residues, is of great importance. This is particularly significant in the context of uncovering the mechanisms of diseases associated with single nucleotide polymorphisms (SNPs), and consequently, developing effective treatments.

**Methods:**

Two methodological approaches were applied to study the complexation behavior. In the first, ligand and metal ions were mixed at low pH, and complexes formed upon gradual pH increase (via NaOH addition). The formation of different complex forms at changing pH was observed, and stability constants were determined. Spectroscopic data allowed prediction of coordination modes linked to structural changes. The second approach involved complex formation in buffered solutions at fixed pH. Here, metal ion solution was added to partially deprotonated ligands, potentially influencing the complexation behavior compared to the first method.

**Results and Discussion:**

This study highlights the importance of His-131 and Glu-126 residues in Cu^2+^ and Zn^2+^ ion binding by peptide fragments of the HSPB1 protein. These residues are essential for both the stability of the complexes and the nature of their interaction with the metal ions. Analytical methods exploring complexation behavior across a pH range of 2–10 and in buffer solutions provide a comprehensive view of the thermodynamic properties of the studied systems. This enables the prediction of their behavior under diverse conditions.

## 1 Introduction

Almost one-third of all known proteins bind metal ions (Ibers and Holm, 1980; Tainer et al., 1991). Metal ions play a crucial role in the structure and function of such proteins, as their biological function depends on the interaction between the ligand-binding residues and the metal ions (Tupler et al., 2001; Caputo et al., 2008). The general molecular mechanism involves the binding of metal ions to specific residues within proteins, and usual binding residues are Cys, His, Glu, and Asp (Babor et al., 2008; Dutta and Bahar, 2010). Changes to these residues–often due to genetic mutations–can alter binding affinity and protein function. Single nucleotide polymorphisms (SNPs), occurring in over 1% of the population, can replace a single nucleotide, potentially altering the encoded amino acid and affecting protein-metal interactions. Such changes may contribute to disease development (Levy et al., 2011; Conde et al., 2006). Identifying metal ion binding sites is thus crucial for understanding protein function, and also for rational drug design targeting mutation-induced dysfunctions.

SNPs are a valuable tool in genetics for identifying disease-associated genes (Conde et al., 2006). For instance, congenital cataracts, a major cause of childhood blindness (Ge et al., 2014), are linked to mutations in the *CRYAA* and *CRYAB* genes (Jarwar et al., 2022; Cui et al., 2017), which encode small heat shock proteins (sHSPs) involved in maintaining lens transparency. These proteins form α-crystallin oligomers that interact with metal ions; such interactions stabilize the oligomers and preserve optical clarity (Karmakar and Das, 2011). Mutations that disrupt these interactions can promote cataract formation.

sHSPs are highly conserved across species and act as molecular chaperones, mitigating protein misfolding under stress. They are implicated in neurodegenerative disorders, cancer, and congenital diseases, making them attractive therapeutic targets (Treweek et al., 2015; Bakthisaran et al., 2015). These proteins are also upregulated in response to environmental stressors like heavy metals (Szebesczyk and Slowik, 2023), which can disrupt protein folding. HSPs help preserve cellular proteostasis under such conditions (Zhang et al., 2013). Increasing ROS levels due to metal ion exposure also trigger HSP expression, contributing to cellular defense (Szebesczyk and Slowik, 2023).

The impact of metal ions is context-dependent, varying by organism, tissue, and interacting molecule. In α-crystallin, Zn^2+^ ions increase the thermodynamic stability of oligomers (Karmakar and Das, 2011). These oligomers–composed of CRYAA (HSPB4/αA-crystallin) and CRYAB (HSPB5/αB-crystallin) – have an average molecular weight of ∼800 kDa and prevent protein aggregation in the lens during thermal stress (Maulucci et al., 2011). Point mutations in these genes are linked to non-syndromic, hereditary cataracts (Andley, 2009). Given the lens’s lifelong function, α-crystallin must retain high structural stability.

Metal ion exposure has been identified as a cataract risk factor. Although typical metal concentrations in the lens range from 0.4 to 30 μg/g tissue (González-Iglesias et al., 2017), higher levels have been observed in cataract patients (Dawczynski et al., 2002; Aydin et al., 2005). Metal ions like copper and zinc induce γ-crystallin aggregation, whereas interactions with β-crystallins are less understood (Ramirez-Bello et al., 2022). Investigating these interactions is technically challenging and expensive, requiring multiple analytical techniques (Witkowska and Rowinska-Zyrek, 2019). Peptide fragments can serve as models to facilitate identification of metal-binding residues and to assess how mutations influence binding geometry and complex stability.

It was found that the Zn^2+^-dependent inter-subunit bridge increases the thermodynamic stability of α-crystallin oligomers and makes them resistant to dissociation even in 6 M urea solutions, while the zinc ions themselves can be removed by dialysis (Karmakar and Das, 2011). In further studies, Karmakar et al. found that the Zn^2+^ binding residues in α-crystallin are His residues, i.e., H79, H107 and H115 of CRYAA and H104, H111 and H119 of CRYAB (Karmakar and Das, 2012). Therefore, age-related deficiency of Zn^2+^ ions may lead to a decrease in the stability of α-crystallin oligomers and may be a risk factor for the development of cataracts. Furthermore, results of the *in vitro* experiments showed that bivalent zinc interacts specifically with α-crystallin, with a dissociation constant in the submillimolar range (Kd ∼ 0.2–0.4 mM). The addition of 1 mM Zn^2+^ increased the yield of α-crystallin-assisted refolding of chemically denatured protein, specifically urea-treated LDH (lactate dehydrogenase), to its native state from 33% to 38%. The surface hydrophobicity of α-crystallin was increased by 50% with the binding of Zn^2+^ (Biswas and Das, 2008).

Of all the bivalent metal ions tested, Zn^2+^ showed the strongest effect on the structure of CRYAA/CRYAB, structural proteins that are essential for the optical properties of the lens. Already at a concentration of 1 mM it can significantly increase the chaperone activity of these proteins (Biswas and Das, 2008). Conformational studies showed that the presence of Zn^2+^ does not change the secondary and tertiary structures of CRYAA/CRYAB, but increases the hydrophobicity. Mass spectrometry studies with diethyl pyrocarbonate (DEPC)-modified CRYAA and CRYAB showed that residues His79, His107 and His115 in CRYAA and His104, His111 and His119 in CRYAB bind to Zn^2+^ (Karmakar and Das, 2012). All of these histidine residues are located in the β5-β6+7 strands of ACD and most likely form a bridge between the subunits with the Zn^2+^ ion, making the dimer structure more stable. The human lens contains approximately 20 μg of zinc per Gram of dry lens tissue (Grahn et al., 2001), but it is unknown how much of this is actually associated with CRYAA/CRYAB.

The binding of Cu^2+^ by CRYAB has also been investigated (Ganadu et al., 2004; Mainz et al., 2012). It is hypothesized that the N-terminal and middle domain residues, namely, His101, His119, Lys121, His18 and Glu99 (Lys and Glu complete the coordination sphere) are able to anchor and bind Cu^2+^. The studies were performed by spectroscopic studies and molecular modeling (Ganadu et al., 2004). His residues are common for Cu^2+^ complex formation in many proteins (Migliorini et al., 2010; Schilling et al., 2020; Smith et al., 2008; Prabhu et al., 2011). In 2011, seven peptide fragments of diethylpyrocarbonate modified CRYAB with potentially Cu^2+^-binding histidine residues were identified by on-column trypsinization followed by MALDI-TOF mass spectrometry, with 3 residues found in the N-terminal domain and 4 in the C-terminal domain (Prabhu et al., 2011). Subsequently, NMR studies showed that His and Asp residues, namely, H83, H104, H111 and D109 at the dimer interface, are involved in the formation of a copper complex with picomolar binding affinity (Mainz et al., 2012). This model showed similarity to the Zn^2+^ binding model. In addition, the sequence of amino acids in the binding loop is also conserved in human CRYAA and HSPB1. All sHSPs contain a conserved domain in their structure, so-called the α-crystalline domain. It is involved in dimer formation and as such plays an extremely important role in maintaining protein chaperone function (Bagneris et al., 2009; Baranova et al., 2011; Muranova et al., 2019; Mymrikov et al., 2012; Delbecq et al., 2015; Heirb et al., 2017).

**FIGURE 1:**
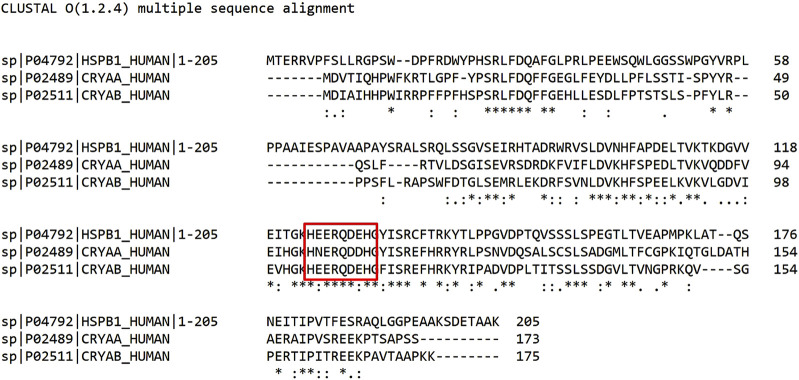
Sequence alignment of HSPB1, α-A crystallin (CRYAA), and α-B crystallin (CRYAB). The studied peptide consists of the amino acid residues 124–131 of HSPB1, 104–111 residues of CRYAB, and is similar to 100–107 residues of CRYAA (evidenced in the red box). Created by UniProt (www.uniprot.org).

In this study, we investigated the coordination properties of an 8-residues fragment of the conserved α-crystallin domain of the heat shock protein β-1 (HSPB1, Figure 1). The same octapeptide sequence is also found in α-B-crystallin (CRYAB) and, similar, in α-A-crystallin (CRYAA, Figure 1).

The impact of specific coordinating amino acids of this peptide on the formation of copper and zinc complexes was investigated by designing analogs of the native peptide in which a selected unit(s) were substituted with alanine, whose side chain does not possess complexometric propensities.

**FIGURE 2:**
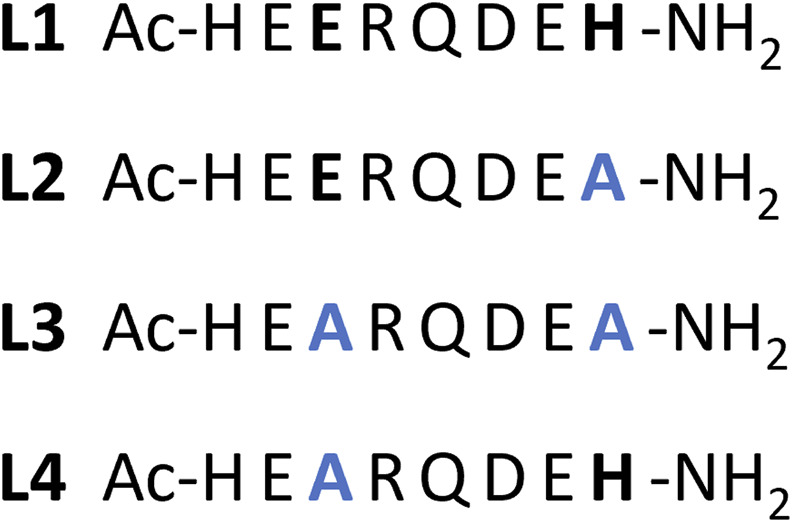
Schematic representation of the studied peptides with the position of the aminoacidic substitutions evidenced in bold black → blue in their structure. L1 – Ac-HE**E**RQDE**H**-NH_2_; L2 - Ac-HE**E**RQDE**A**-NH_2_; L3 - Ac-HE**A**RQDE**A**-NH_2_; L4 - Ac-HE**A**RQDE**H**-NH_2_.

Specifically, substitution with non-coordinating alanine residues was used to test whether the Glu-126 and His-131 residues are crucial for metal ion coordination (Figure 2). L1 has the native sequence, whereas L2-L4 are the designed analogs.

The use of short peptides allowed to isolate and characterize local binding events that would be more difficult to resolve in the context of the full-length, flexible, and oligomeric protein. While this approach does not replicate full protein behavior, it provides a focused view of potential coordination motifs.

## 2 Materials and methods

All peptides, i.e., Ac-HEERQDEH-NH_2_, Ac-HEERQDEA-NH_2_, Ac-HEARQDEA-NH_2_, and Ac-HEARQDEH-NH_2_, were purchased from KareBay Biochem (Monmouth Junction, NJ, USA) and were used as received (certified purity >95%). All solutions were prepared in ultrapure deionized water (Polwater DL-3, Krakow, Poland) equipped with a UV lamp and 0.22 µm filter with a maximum conductivity of 0.06 μS/cm. Stock solutions were prepared using a Mettler Toledo analytical balance with an accuracy of 0.01 mg. The solution of HCl (Chempur, Piekary Śląskie, Poland) in KCl (Chempur) was titrated with standardized 0.1 M NaOH (0.1 M NaOH concentrate from Sigma-Aldrich, Poznan, Poland). Carbonate-free NaOH solution (Sigma-Aldrich 0.1 M NaOH concentrate) was standardized by titration with potassium hydrogen phthalate (Sigma-Aldrich).

### 2.1 Potentiometric titration

The potentiometric titrations were carried out using an Omnis automatic titrator system (Metrohm, Opacz-Kolonia, Poland) equipped with a combined Biotrode® glass electrode with Idrolyte® filling. The ionic strength was set to I = 0.1M with KCl (Chempur). The combined glass electrode was calibrated as a probe for hydrogen concentration by titrating known amounts of HCl (0.004M) with carbonate-free NaOH solution (0.1M). A stream of argon pre-saturated with water vapor flowed over the surface of the solution cell, which was filled with 3.0 mL of the solution under investigation and thermostatted to 25°C ± 0.2 °C. For the potentiometric measurements, the c_lig_ = 0.001M, the ratio of metal to ligand was 1:1 and 1:2. The concentrations of the peptides were determined by potentiometric titration of the ligands. After careful concentration calculation for each ligand, a sample was taken from the same stock solution for potentiometric measurements of the complex. The peptide solution was freshly prepared prior to the measurements of the ligand, in an amount sufficient to titrate the ligand and subsequently the complex. The concentration of a stock solution of metal ions was determined by ITC titration with EDTA solution of known concentration. Approximately 150 data points were collected for the complexes. The potentiometric data were evaluated using the Hyperquad 2013 program (Gans et al., 1996). The distribution and competition diagram was calculated using the program HYSS (Alderighi et al., 1999).

### 2.2 ITC–Isothermal titration calorimetry

Isothermal titration calorimetry (ITC) measurements were performed at 25°C on a MicroCal PEAQ titration calorimeter (Malvern, United Kingdom). All reagents, except KCl, were >99% pure and were purchased from Sigma-Aldrich. Peptides were dissolved directly in a 0.1M buffer solution (MES, Sigma Aldrich), the pH of which was adjusted to 6.1 with NaOH or HCl. Metal stock solutions (copper (II) chloride, 50 mM; zinc(II) chloride, 50 mM) were prepared in deionized water (maximum conductivity of 0.06 μS/cm) at low pH (∼2). After stabilization of the device at 25°C, 40 µL of a metal buffer solution (2 mM) was used to titrate 200 µL of a peptide buffer solution whose concentration was initially ten times lower than that of the metal ion (i.e. 0.2 mM). Each titration consisted of 19 or 26 consecutive injections with an interval of 180–240 s between each aliquot (depending on the time required for complete equilibration) and a stirring speed of 750 rpm, repeated at least three times. The heat of dilution was subtracted from each injection. An initial injection of 0.4 µL was removed from each data set to eliminate the effect of diffusion of the titrant through the syringe tip during the equilibration process (Wyrzykowski et al., 2013). A CaCl_2_–EDTA titration was performed at regular intervals for comparison with the results of the initial calibration of the device. Data were processed using MicroCal PEAQ-ITC Analysis Software. Blank subtraction (peptide titration into buffer) was consistently performed for all ITC experiments. The one-side binding model provided the best-fitting values for stoichiometry (n), enthalpy change (ΔH), and a binding constant (Kd).

### 2.3 UV-vis titration spectroscopy

Absorbance spectra were recorded with the NanoDrop OneC spectrophotometer (ThermoScientific) in the range of 200–800 nm and pH 2–10 using a quartz cuvette with an optical path of 1 cm. The pH changes were determined by adding HCl or NaOH using a pH meter (Mettler Toledo) equipped with a combined glass electrode (InLab Semi-micro) with polymer filling (XEROLYT® EXTRA). Similarly to the potentiometric titration, the concentration c_lig_ = 0.001M, the ratio of metal to ligand was 1:1 and 1:2. Samples were prepared in a vial, then moved to the quartz cuvette with a 10 mm optical path length. The pH was changed by the addition of small drops of concentrated HCl or NaOH, to avoid volume changes.

### 2.4 CD spectroscopy

Circular dichroism (CD) measurements were performed with a Jasco J-715 CD spectropolarimeter. The spectra were recorded in the 800–230 nm range using quartz cuvettes with an optical path of 1 cm. The peptide solutions were prepared in a 0.1 M HEPES buffer with a pH of 6.1. The concentration of each peptide was 0.1 mM. Spectra were recorded after the addition of 0–1 equivalents of Cu(II) or Zn(II) with 0.1 steps. Exceeding the ratio of 1:1 resulted in the precipitation of copper hydroxide.

The pH titration of the complexes formed at pH ∼2 was also performed. The metal ions concentration was 0.05 mM and ligand concentration 0.1 mM, in a water solution containing 0.1 M KCl, and c_lig_ = 0.00035M with metal to ligand ratio 1:2 for far-UV studies in the 180–300 nm range. The studies were performed in a 10 mm (300–800 nm) and 0.1 mm (180–300 nm) quartz cuvettes. Samples were prepared in a vial, where the pH was changed using small drops of concentrated HCl or NaOH, and before measurement in far UV, 120 µL was transferred to the cuvette.

Further parameters were calculated using the dedicated JASCO Spectra Manager programs and graphs were visualized using Origin software. The equation used for calculation of Δε is given below.

$$\Delta \varepsilon \;\left(M^{-1}\times {cm}^{-1}\right)=\frac{\theta }{\left(32980\times c\times l\right)}$$

where θ is the CD ellipticity in millidegrees, c is concentration in mol/L, and l is the path length in cm.

## 3 Results

### 3.1 Acid-base properties of the studied peptides

Since the studied peptides included amino acids with side chains containing proton-releasing groups, we determined the deprotonation constants (acid dissociation constant, *K*_a_) for such groups. The results summarized in Table 1 show that some of the protons belonging to the carboxylic groups of Glu and Asp are difficult to distinguish. In addition, L1 has 6 possible deprotonating groups in the structure, but only five constants were determined, most likely due to the superposition of some carboxylic acid *K*_a_ values.

**TABLE 1:** Acid dissociation constants determined for the investigated L1-L4 peptides. Potentiometric titrations were performed with aq. NaOH by increasing the pH over the range 2–11 at T = 25°C in an aqueous solution containing 4 mM HCl, 0.1 M KCl and 0.001 M concentration of each peptide. Residues with side chain groups that can deprotonate are underlined. The standard deviations are given in brackets as uncertainties for the last significant digit. *β* is the stability constant of given complex form.

	*L1 - Ac-HEERQDEH-NH*_*2*_		*AA assigned*	*L2 - Ac-HEERQDEA-NH*_*2*_		*AA assigned*
Form	log*β*	p*K*_a_		log*β*	p*K*_a_	
LH	7.20 (1)	7.20	His	7.00 (1)	7.00	His
LH_2_	13.32 (1)	6.12	His	12.15 (2)	5.15	Glu
LH_3_	17.77 (2)	4.45	Glu	16.62 (2)	4.47	Glu
LH_4_	21.78 (2)	4.01	Glu	20.50 (2)	3.88	Glu
LH_5_	25.08 (2)	3.30	Glu/Asp	24.10 (2)	3.60	Asp

	*L3 - Ac-HEARQDEA-NH*_*2*_		*AA assigned*	*L4 - Ac-HEARQDEH-NH*_*2*_		*AA assigned*
Form	log*β*	p*K*_a_		log*β*	p*K*_a_	
LH	6.71 (1)	6.71	His	7.24 (4)	7.24	His
LH_2_	11.51 (1)	4.80	Glu	13.66 (2)	6.42	His
LH_3_	15.57 (1)	4.06	Glu	18.40 (4)	4.74	Glu
LH_4_	19.04 (2)	3.47	Asp	22.34 (3)	3.94	Glu
LH_5_				25.60 (3)	3.26	Asp

The highest p*K*_a_ values, i.e. 7.20 and 6.12 for L1, 7.24 and 6.42 for L4, 7.00 for L2, and 6.71 for L3, can be assigned to the deprotonation of the imidazole N-3 of the His residues, as the values are in reasonable agreement with literature data for this process (Migliorini et al., 2010; Watly et al., 2018; Hecel et al., 2020).

Other p*K*_a_ values, from the lowest one at 3.26 of L4 to the highest at 5.15 of L2, can be assigned to the deprotonation of carboxylic groups (Rola et al., 2022). However, it was not possible to determine from the potentiometric titration which value corresponds to Glu or Asp residues for L1 due to the overlapping of the deprotonation processes.

### 3.2 Evaluation of metal complex binding

#### 3.2.1 Potentiometric and UV-vis titration

**FIGURE 3:**
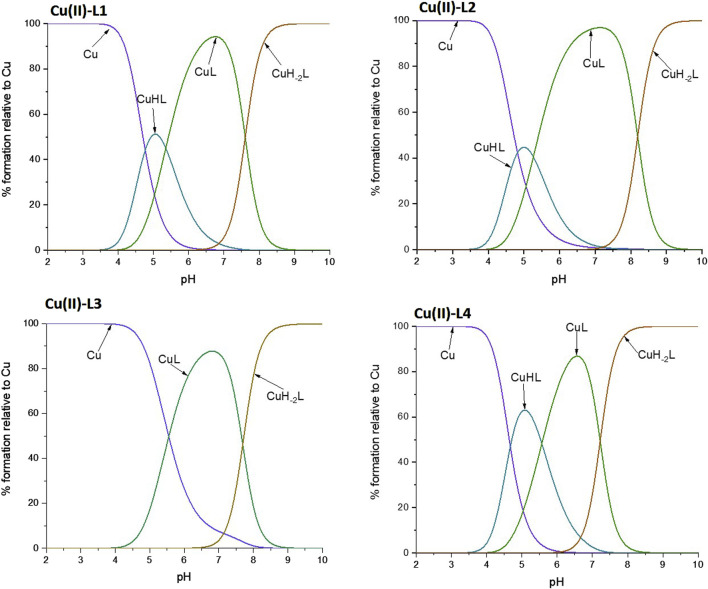
Schematic distributions of the complex forms for the investigated ligands with copper (II) ions. The Cu(II) concentration used for the simulation was 1·10^-4^ M, and the metal to ligand ratio was 1:10.

The ability of the studied peptide ligands to form copper and zinc complexes was evaluated by potentiometric titration in the pH range 2–10 using a fixed metal-to-ligand ratio (1:2 and 1:4). Since the Cu(II) complexes were already formed at a pH of about 5 (at least for L1, L2 and L4, Figure 3), it was possible to determine their stability constants relatively to the copper coordination of the partially protonated ligands. The best fit between the experimental and theoretical curves was obtained for the stability constant values presented in Table 2. Based on these stability constants, a species distribution diagram was constructed (Figure 3) to illustrate the relative concentrations of complex forms as a function of pH, with reference to the percentage content of copper ions in solution.

**TABLE 2:** Stability constants of the Cu(II) and Zn(II) complexes with the studied peptide ligands. Titrations were performed over the pH range 2–11 at T = 25°C in an aqueous solution containing 4 mM HCl and 0.1 M KCl. The peptide concentration was 0.002 M with a metal-to-ligand ratio of 1:2 and 1:4 for UV-vis pH titrations, and 0.001 M with a metal-to-ligand ratio of 1:2 for CD titrations. N is the number of metal-coordinating nitrogen atoms.

Complex form	L1		UV-vis			CD	
	Log*β*	p*K*_a_	λ [nm]	ε [M^-1^cm^-1^]	N	λ [nm]	Δε [M^-1^cm^-1^]
CuHL	11.79 (6)	5.35	690	40	1	465 255	0.25 0.44
CuL	6.44 (7)		655	70	2	493 255	0.43 4.60
CuLH_-2_	−8.77 (9)		603	110	3	494 412 318	−0.83 0.08 0.62
ZnL	4.00 (6)						

Complex form	L2		UV-Vis			CD	
	log*β*	p*K*_a_	λ [nm]	ε [M^-1^cm^-1^]	N	λ [nm]	Δε [M^-1^cm^-1^]
CuHL	10.65 (4)	5.26	686	30	1	447	0.08
CuL	5.39 (5)		655	50	2	527 439 311 260	−0.57 0.09 0.41 2.28
CuLH_-2_	−10.99 (7)		585	120	3	527 309 262	−1.61 1.56 5.58
ZnL	2.7 (1)						

Complex form	L3		UV-Vis			CD	
	log*β*	p*K*_a_	λ [nm]	ε [M^-1^cm^-1^]	N	λ [nm]	Δε [M^-1^cm^-1^]
CuHL							
CuL	4.28 (7)		682	20	1	447 348 302 255	0.09 0.11 −0.40 0.60
CuLH_-2_	−11.10 (8)		633	70	2	526 315 260	−1.11 0.56 3.75
ZnL	n/a						

Complex form	L4		UV-Vis			CD	
	log*β*	p*K*_a_	λ [nm]	ε [M^-1^cm^-1^]	N	λ [nm]	Δε [M^-1^cm^-1^]
CuHL	12.42 (5)	5.56	680	50	1	456 253	0.25 0.77
CuL	6.86 (8)		633	80	2	470 355 255	0.12 −0.23 4.67
CuLH_-2_	-7.58 (9)		606	120	3	496 319 258	−0.92 0.72 6.51
ZnL	4.09 (2)						

Despite attempts to include complex species with metal-to-ligand ratios other than 1:1 in the model, it was not possible to obtain results with acceptable error values for such species. This suggests that these forms either do not form under the experimental conditions or are present in concentrations too low to be reliably detected using the employed analytical techniques.

pH-Titration was also monitored by UV-vis spectroscopy and the obtained results were compared with the potentiometric data. In this way, we were able to associate a specific constant of a complex with the specific binding mode (Table 2). L1 and L4 showed a similar complexation behavior towards Cu(II), exhibiting the highest stability constants, while L3 formed the least stable complex. The trend was also the same for the Zn(II) complexes, but the stability constant of Zn(II)-L3 could not be determined by potentiometric titration since the interaction is weak and hydrolytic forms of zinc predominate in solution. UV-Vis spectroscopy was not used for studies of Zn(II) complexes, as this metal ion is spectroscopically silent.

For all four peptides, the complexation behavior appeared to be similar, i.e., it starts from a 1N coordination (one nitrogen atom involved in the coordination of the copper ion), followed by a 2N coordination, then the deprotonation of the amide leads to a 3N coordination mode. Such changes in the number of coordinating nitrogen atoms are proven by the blue shift to characteristic values of the wavelength maximum (Supplementary Table S5), along with the increase in absorbance, confirming the complex formation. The metal-to-ligand stoichiometry of the complexes was found to be 1:1. However, the nitrogen atoms involved differ depending on the ligand. In the case of L1 and L4, the proposed coordination mode starts with the nitrogen of the imidazole ring of a His residue (N_im_)), then a second N_im_ coordinates the Cu(II) ion. However, to stabilize the structure of the complex, the deprotonation of two amide backbone nitrogen atoms takes place. Thus, finally, only one N_im_ together with two N^−^ (from the amides) are involved in the final 3N complex. In L2, which has only one His, complex formation begins with the anchoring of the Cu(II) ion to that His residue. Then, the forced deprotonation of two amides leads to the stabilization of the complex, which is completed again by the formation of a 3N coordination system. Considering the reduction of the stability constant (log*β*) for the Cu-L2 complex by an order of magnitude compared to Cu-L1, it can be concluded that His-131 (Figure 2) is of great importance for the formation and stability of the complex. In addition, the stability constant of Cu-L3 is reduced by an order of magnitude compared to Cu-L2, indicating also the importance of the Glu-126 residue (Figure 2) for the stabilization of the complex. These hypotheses are also confirmed in the case of Zn(II) complexes. The stability constants for Zn-L1 and Zn-L4 are very similar, but the absence of His-131 in L2 leads to a decrease in logβ from about 4 to 2.7, which is more than an order of magnitude. The influence of Glu-126 is even more pronounced in the case of the zinc complexes, as the Zn-L3 complex is not formed in sufficient concentration to obtain reliable calculation results due to the prevailing hydrolysis process.

From these studies, it can be concluded, that His-131 is the most important residue in metal ion binding, and the lack of both His-131 and Glu-126 results in decreased complex stability (for the Zn-L3 system the complex was formed in insufficient amounts to determine stability constants by potentiometric titration).

#### 3.2.2 ITC experiments

Calorimetric measurements of the binding of metal ions to ligands, peptides and proteins offer the possibility to determine the enthalpic and entropic origin of the free energy of complex formation (stability). However, Isothermal Titration Calorimetry (ITC) measures the net thermodynamics of the binding (it provides the condition-dependent thermodynamic values) (Wyrzykowski et al., 2013).

In this study, it was possible to compare the ITC results of all four peptides analyzed, as all experiments were carried out under the same conditions (pH, temperature and buffer).

**FIGURE 4:**
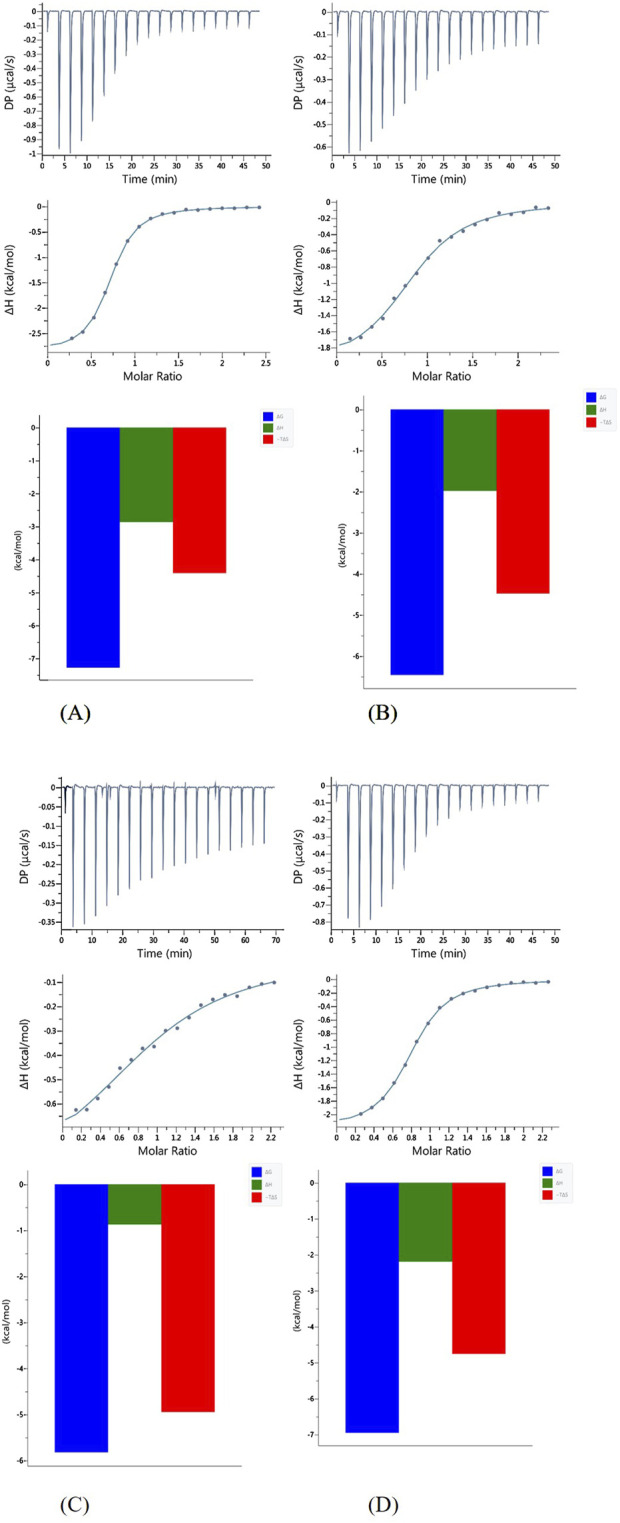
Representative ITC data (above) and their corresponding thermodynamic signatures (below) for Cu(II) titrated into **(A)** L1 Ac-HEERQDEH-NH_2_, **(B)** L2 Ac-HEERQDEA-NH_2,_ **(C)** L3 Ac-HEARQDEA-NH_2,_ and **(D)** L4 Ac-HEARQDEH-NH_2_ peptides at pH 6.1.

The results of ITC experiments also proved the importance of His-131 in metal ion coordination. Indeed, peptides in which the His-131 residue was substituted by Ala (L2 and L3) showed a much weaker affinity (K_dITC_ is about one order of magnitude lower) for Cu(II) ions than L1 and L4 (Figure 4; Table 3).

**TABLE 3:** Results of ITC experiments for all of the studied ligands with Cu(II) ions at T = 25°C.

	Cu(II)-L1	Cu(II)-L2	Cu(II)-L3	Cu(II)-L4
N	0.595 ± 0.0021	0.625 ± 0.0070	0.684 ± 0.028	0.605 ± 0.0033
KD (M^-1^)	(4.81 ± 0.17)x10^6^	(2.16 ± 0.17)x10^5^	(4.94 ± 0.13)x10^5^	(7.57 ± 0.40)x10^6^
ΔH (kcal/mol)	−2.87 ± 0.019	−2.065 ± 0.052	−0.827 ± 0.089	−2.30 ± 0.026
ΔG (kcal/mol)	−7.25	−6.37	−5.87	−6.99
TΔS (kcal/mol)	−4.38	−4.31	−5.05	−4.69

All reactions are enthalpy- and entropy-driven, as evidenced by the favorable negative enthalpy change and the positive entropy contribution. This suggests that the coordination of Cu(II) by the peptide is stabilized by specific enthalpic interactions, such as metal–ligand bonding, as well as by favorable entropic effects, likely resulting from desolvation of the metal ion and/or conformational changes in the peptide upon complex formation. Although both enthalpic and entropic contributions are favorable, Cu(II) coordination by the peptides appears to be predominantly entropy-driven, with desolvation and conformational effects likely contributing to the larger positive TΔS value. Although in the case of the binding of L3 to Cu(II), the effect of entropy change predominates (Figure 4), which could be due to the presence of two Ala residues in the sequence and resulting in different interactions involved in metal complex formation. Measurements carried out in a buffered solution showed n ≈ 0.6, suggesting that the stoichiometry was 1:2 (one metal ion bound by two peptides). Such behavior was observed earlier for other systems (Witkowska et al., 2021; Miller et al., 2021). In this case, the different stoichiometry compared to the potentiometric studies could be because at the investigated pH of 6.1, the His residues are at least partially protonated, so the first step of complexation could be the electrostatic interaction between the positively charged Cu (II) and the negatively charged deprotonated carboxyl groups of the Glu and Asp side chains. Subsequently, the proximity of His-131 contributes to obtaining a thermodynamically stable complex.

**FIGURE 5:**
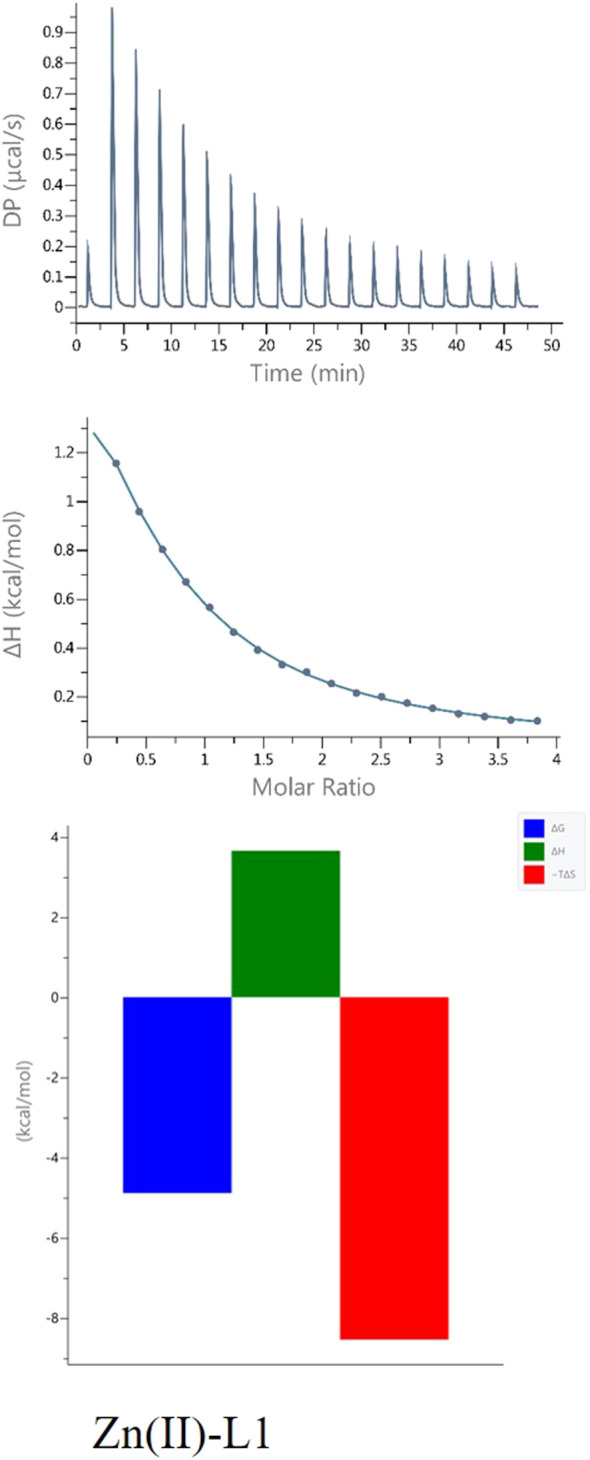
ITC data (above) and their corresponding thermodynamic signatures (below) for Zn(II) titrated into L1 Ac-HEERQDEH-NH_2_.

**FIGURE 6:**
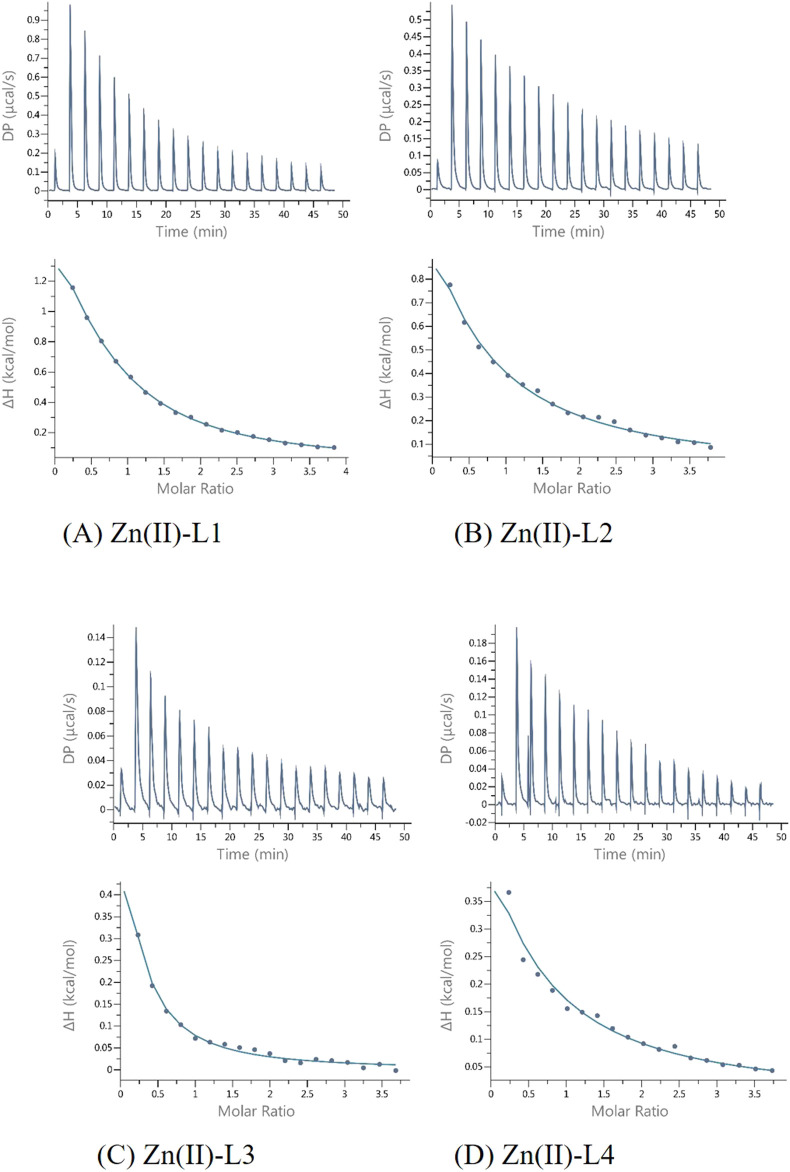
Far-UV CD results for L1-Cu(II) system; **(A)** Titration of ligand; **(B)** Titration of complex with Cu(II); **(C)** Comparison of ligand and complex spectra in pH = 7.

The L1-Zn complex also shows a stoichiometry of 2:1 (n ≈ 0.5) and there is a large entropic contribution to this reaction (-TΔS = − 8,08, Figure 5). The Zn^2+^ binding to the other peptides (L2, L3 and L4) was too weak to allow the parameters calculations with reasonable errors (Figure 6) (Turnbull and Daranas, 2003). This confirms the importance of both His-131 and Glu-126 for the coordination of this metal ion in a certain pH conditions.

#### 3.2.3 CD analysis

The CD analysis was performed following two different approaches. In the first set of experiments, the selected peptide was incubated with Cu (II) at a metal-to-ligand ratio of 1:2 at pH 2, and then the pH was increased with NaOH titration in steps of 0.5–1, up to 10 on the pH scale. From these experiments, we can determine the Δε values, included in Table 2, as characteristics for certain complex forms. Also, the far-UV experiments were performed with a similar approach.

**FIGURE 7:**
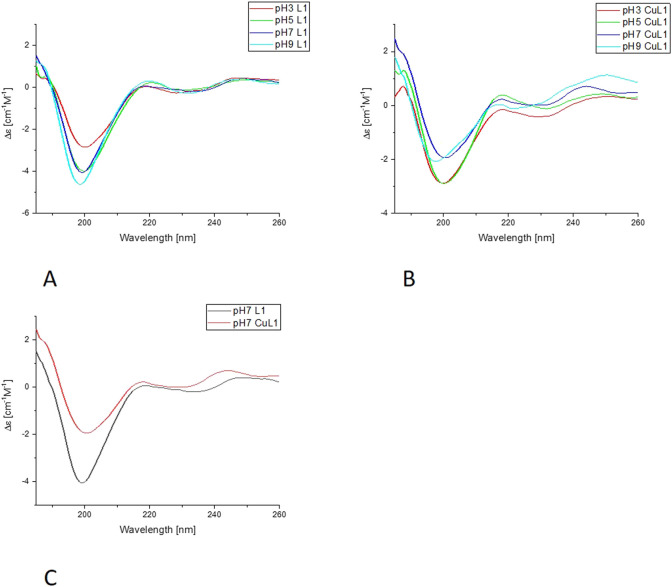
Far-UV CD results for L4-Cu(II) system; **(A)** Titration of ligand; **(B)** Titration of complex with Cu(II); **(C)** Comparison of ligand and complex spectra in pH = 7.

**FIGURE 8:**
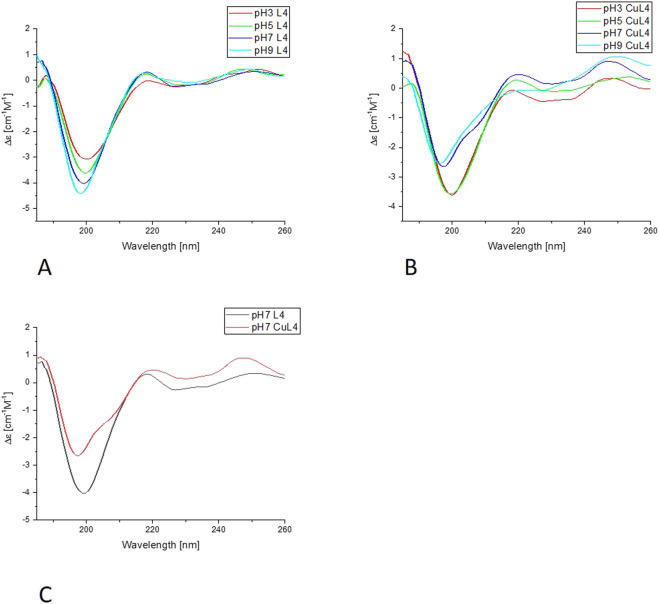
Representative ITC data (thermograms) for Zn(II) titrated into **(A)** L1 Ac-HEERQDEH-NH_2_, **(B)** L2 Ac-HEERQDEA-NH_2,_ **(C)** L3 Ac-HEARQDEA-NH_2,_ and **(D)** L4 Ac-HEARQDEH-NH_2_ peptides at pH 6.1.

In the far-UV CD experiments, the random coil characteristic spectrum is observed for all ligands (Figures 7, 8, Supplementary Figures S4, S5), with a negative band at around 200 nm. Increasing the pH results in a slight blue shift and an increase in the intensity of this band. The addition of Cu(II) results in a decrease in the intensity of the band around 200 nm for all of the systems. The changes are more pronounced for L1 and L4 copper complexes. Nevertheless, for L2 and L3, the blue shift of the band around 200 nm is also very clear, as well as some changes in the intensity of the positive peak at 218 nm, especially for L3. For all of the studied systems, the greatest changes are visible with the pH increase from 7 to 9. The increase of the intensity of the positive band at around 218 nm, along with the decrease of the negative band at 200 nm, indicates the changes in the structure of the complexes, but it still remains loose in terms of secondary structure possibilities. The changes may be caused by the involvement of amides in metal ion binding, enabling the electrostatic interaction or hydrogen bonds. The similarities in the behavior of pairs of ligands, L1 and L4, and L2 and L3, may be caused by the presence or absence of His-131 and the different anchoring sites for Cu(II) ion binding.

**FIGURE 9:**
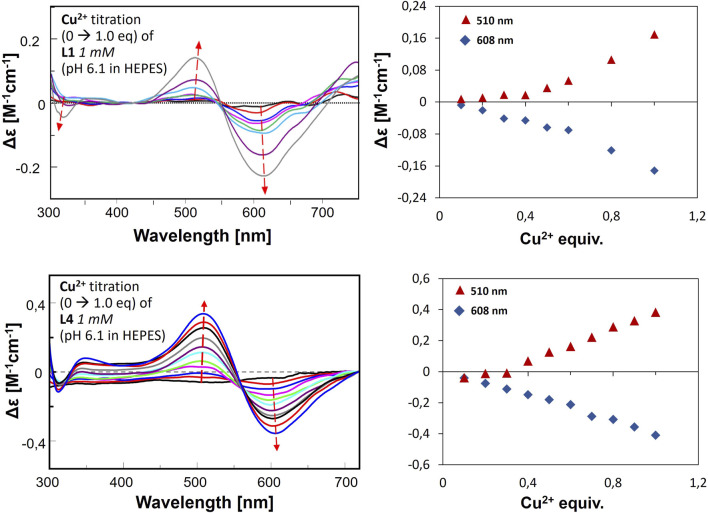
CD spectra for metal ion titration in buffer, L1-Cu(II) (upper graph) and L4-Cu(II) (lower graphs). The right panel presents the changes of Δε by certain wavelengths according to Cu(II) equivalent titrated into the ligand solution.

In the second approach, the ligand was dissolved in a buffered solution at pH 6.1, and an increasing amount of Cu(II) was added in 0.1 equivalent aliquots up to a ratio of 1:1 or slightly higher, 1:1.1. Precipitation occurred with further addition of the Cu(II) solution. Changes in ellipticity, especially in the wavelength 450–700nm, both in positive and negative values for L1-Cu(II) and L4-Cu(II) are presented in Figure 9. In addition, no significant CD signal was observed for Cu-L2 and Cu-L3 in the same spectral region, which could indicate a purely electrostatic interaction (Supplementary Figures S3, S4). Therefore, the presence or absence of His-131 and Glu-126 affects not only the stability of the formed complex but also the nature of the interaction between the ligand and the metal ion.

The results obtained in the second approach for L1-Cu(II) and L4-Cu(II) show transitions at 510 nm (positive) and 608 nm (negative). Two signals (Cotton effect) are related to square-planar geometry, and the increase in their intensity proves the formation of such complexes in greater amounts along with stepwise Cu(II) addition. The results are significantly different from the ones obtained in the titration in the pH range of 2–10, which indicates the formation of various complex forms with the employment of these two methods. Nevertheless, this corresponds to the results of buffer studies conducted using ITC titration.

## 4 Discussion

As already mentioned, two different methodological approaches were used to investigate the complexation behavior of the studied systems. First, ligand and metal ions are mixed at low pH, then complexes are formed at increasing pH by adding small amounts of alkaline solution (NaOH). In this way, we can observe the formation of different complex forms at changing pH and determine the overall stability constants. Together with the use of spectroscopic methods, we can predict the coordination mode associated with the structural changes of the studied systems. The second approach, on the other hand, involves the formation of complexes of ligand and metal ion in a buffered solution, i.e., at a selected pH condition. In contrast to the previous approach, here, small drops of the solution containing metal ions are added to the ligand in a buffered solution at certain pH. Under such conditions the ligands might be partially deprotonated, what may have an impact on the complexation behavior. Such an approach seems to better reflect the conditions prevailing in the cell. It may also lead to different stoichiometries of the complexes formed (Witkowska et al., 2021; Miller et al., 2021), but the tendencies of the more and less stable individuals should be preserved. Regardless of the measurement method, the most stable complexes, with both Cu^2+^ and Zn^2+^, were formed by ligands L1 and L4 – both with a His-131 residue retained. Of the second pair of ligands, L2 and L3, both with substituted His-131 residues, the more stable one possessed the Glu-126 residue. The influence of His-131 and Glu-126 was undeniable in complexes formed at lower pH and in buffered solution, including the influence on the type of interaction between the peptide and the metal ion.

In studies by Prabhu et al., it was found that His-to-Ala modifications of peptide fragments of α-crystallin showed no differences in the stability and significance of the substituted residues (Prabhu et al., 2011). Interestingly, they chose similar fragments with analogous His residues (104-HEERQDEH-111), but only investigated the modification of His-104, which is analogous to His-124 from HSPB1. Their studies were based on molecular models. We did not consider this residue because we were struck by the similarity of the fragment studied to that in a heavy chain of the iron storage protein ferritin – 61-HEEREH-66. In this protein, the residues responsible for the binding of iron ions are Glu-63 and His-66, which we used as guidelines for the modification of the peptide fragment under investigation. The differences in the involvement of the -HE and -EH motifs were part of the motivation for the investigations performed.

**FIGURE 10:**
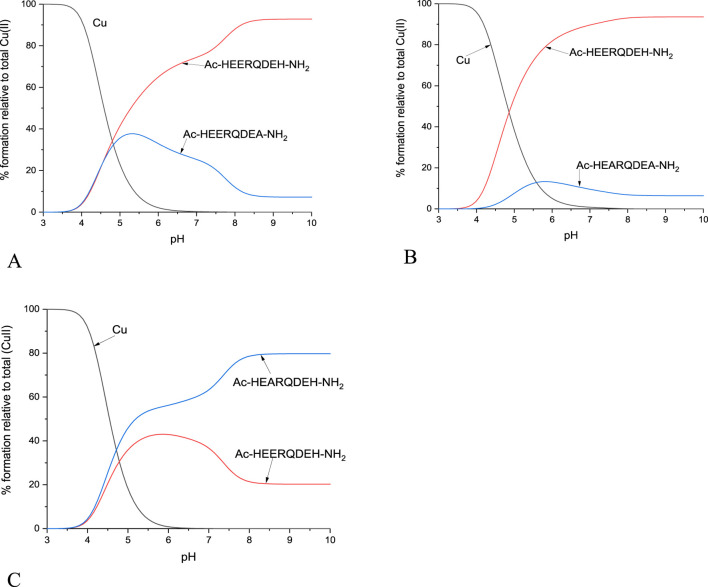
Competition plots for **(A)** L1 and L2, **(B)** L1 and L3, **(C)** L1 and L4 in presence of Cu(II) ions. Previously calculated stability constants are applied to a theoretical situation, in which equimolar amounts of Cu(II) and both ligands are present, in 1:1:1 M ratio.

**FIGURE 11:**
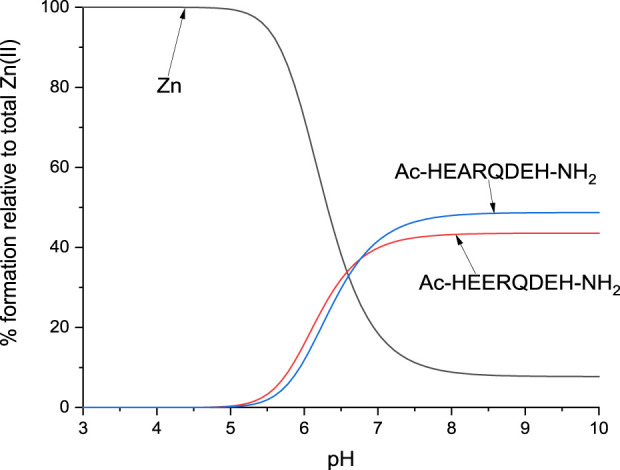
Competition plot for L1 and L4 in presence of Zn(II) ions. Previously calculated stability constants are applied to a theoretical situation, in which equimolar amounts of Zn(II) and both ligands are present, in 1:1:1 M ratio.

In order to present scenarios in which multiple ligands compete for a single metal ion, or a single ligand interacts with multiple metal ions, competitive plots (Figures 10, 11; Supplementary Figure S6) are employed. These diagrams represent a hypothetical situation where equimolar concentrations of ligands and metal ions are present in solution, allowing for a clear comparison of their relative affinities and complexation behaviors (Migliorini et al., 2010; Hecel et al., 2020; Witkowska et al., 2021).

The results of the present study confirm that His-131 plays the most important role in the binding of metal ions and that the absence of His-131 along with the absence of Glu-126 leads to lower stability of the complex, regardless of the condition of the studies, and in buffer, it affects the type of interaction between a ligand and a metal ion. On the other hand, the absence of Glu-126 increases the stability constant of the CuL complex. For Cu(II)-L1, the stability constant is 6.44, while for Cu(II)-L4 it increases to 6.86. Although this difference may not appear substantial at first glance, it becomes much more pronounced when visualized on a competitive plot. Under hypothetical equimolar conditions, L4 exhibits significantly stronger copper-binding affinity and would outcompete other ligands for the metal ion. In the case of zinc ions, the presence of Glu-126 appears to play a less critical role, as evidenced by the competitive plot for L1 and L4 in the presence of Zn(II) ions. The very similar stability constants, 4.00 for Zn(II)-L1 and 4.09 for Zn(II)-L4, indicate comparable binding affinities, which is consistent with the observed plot. In contrast, the absence of His-131 in L2 and L3 resulted in a complete lack of detectable Zn(II) complex formation. This highlights the essential role of this residue in stabilizing Zn(II) coordination.

Interestingly, the complexation behavior in the buffer is similar to that of the whole protein-forming dimers or even larger systems. However, for studied peptides larger aggregates were not observed. Nevertheless, studies are continuing to explain the coordination behavior of -HEE-*versus* -(D/E)EH- motifs in terms of thermodynamic and structural stability.

## Data Availability

The original contributions presented in the study are included in the article/Supplementary Material, further inquiries can be directed to the corresponding author.
