# Supplementary material for: The interaction of Cu(II) and Zn(II) with peptide fragment of HSPB1 and its analogs
Source: Front Mol Biosci. 2025 Sep 4;12:1593661. doi: 10.3389/fmolb.2025.1593661 (PMC12444091; doi:10.3389/fmolb.2025.1593661)
Supplement: Supplementary file 1 [file DataSheet1.pdf]

Supplementary Material

The interaction of Cu(II) and Zn(II) with peptide fragment of HSPB1 and its analogs

Agnieszka Szebesczyk[1,*], Domenica Musumeci[2], Ettore Napolitano[2], Halyna Kukhtenko[1,3], Paulina Iwaniak[4]

[1]Institute of Health Science, University of Opole, Opole, Poland

[2]Department of Chemical Sciences, University of Naples Federico II, Naples, Italy

[3]Department of Cosmetology and Aromology, National University of Pharmacy, Kharkiv, Ukraine

[4]Faculty of Medicine, Medical University of Lublin, Lublin, Poland

* Correspondence:
Agnieszka Szebesczyk
agnieszka.szebesczyk@uni.opole.pl

Supplementary Data

Table S1. UV-Vis spectrum and identified peaks of studied peptide L1 with Cu(II); c_{Cu} = 1mM, metal to ligand ratio 1:4, *T*=25°C, *I*=0,1M KCl.

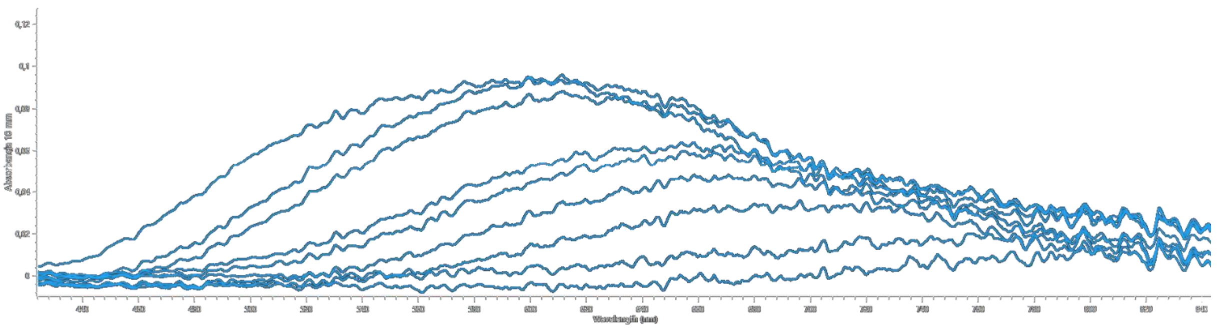

Wavelength [nm]	Absorbance	pH	No. of coordinating N-atoms
800	0,01	3	-
800	0,01	4	-
690	0,03	5	1
670	0,05	6	2

655	0,06	6	2
655	0,06	7	2
613	0,09	8	3
603	0,09	9	3
590	0,09	10	3

Table S2. UV-Vis spectrum and identified peaks of studied peptide L2 with Cu(II); c_{Cu} = 1mM, metal to ligand ratio 1:4, T=25°C, I=0,1M KCl.

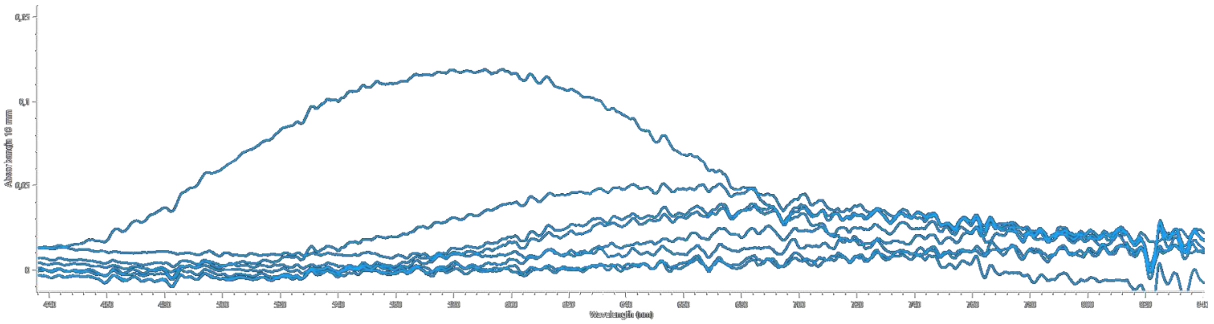

**Wavelength [nm]**	**Absorbance**	**pH**	**No. of coordinating N-atoms**
800	0,01	2	-
800	0,01	3	-
Overlapped max.	0,01	4	1
725	0,03	5	1
686	0,04	6	2
686	0,04	7	2
650	0,05	8	2
585	0,12	10	3

Table S3. UV-Vis spectrum and identified peaks of studied peptide L3 with Cu(II); c_{Cu} = 1mM, metal to ligand ratio 1:4, T=25°C, I=0,1M KCl.

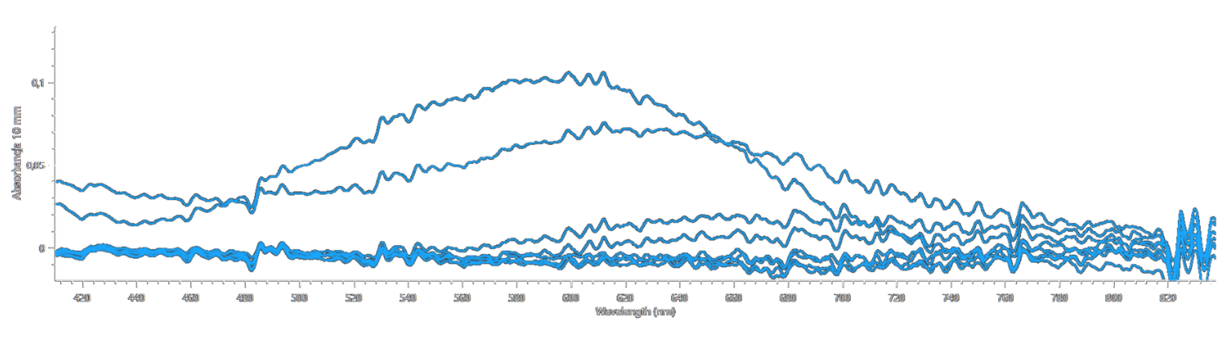

Wavelength [nm]	Absorbance	pH	No. of coordinating N-atoms
800	0,01	2	-
800	0,01	3	-
800	0,01	4	-
800	0,01	5	-
682	0,02	6	1
682	0,02	7	1
633	0,07	8	2
600	0,1	9	2-3 (value between two characteristics)

Table S4. UV-Vis spectrum and identified peaks of studied peptide L4 with Cu(II); c_{Cu} = 1mM, metal to ligand ratio 1:4, T=25°C, I=0,1M KCl.

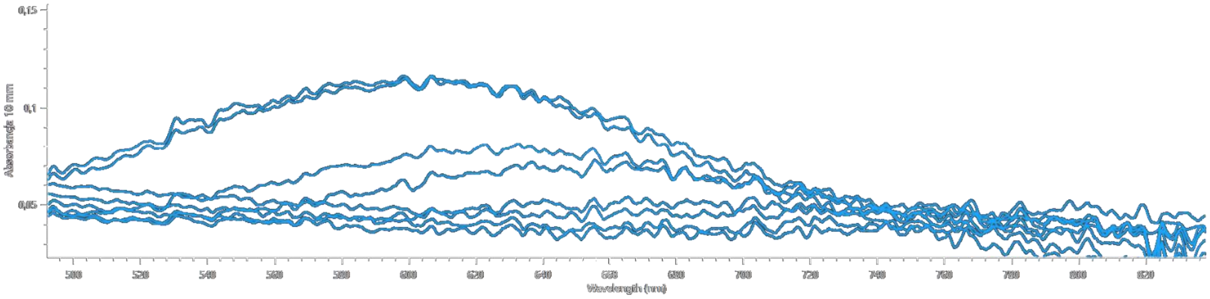

Wavelength [nm]	Absorbance	pH	No. of coordinating N-atoms
780	0,01	2	-
780	0,01	3	-
690	0,04	4	1
687	0,05	5	1
636	0,07	6	2
633	0,08	7	2
606	0,12	8	3
606	0,12	9	3

Table S4. Characteristic values of wavelength maximum depending on the number of N-atoms coordinating copper (II) ion in complexes. [S1]

Number of N-atoms bound to Cu (II)	λ_{max} [nm]
1N	680-730
2N	620-670
3N	540-590
4N	500-530

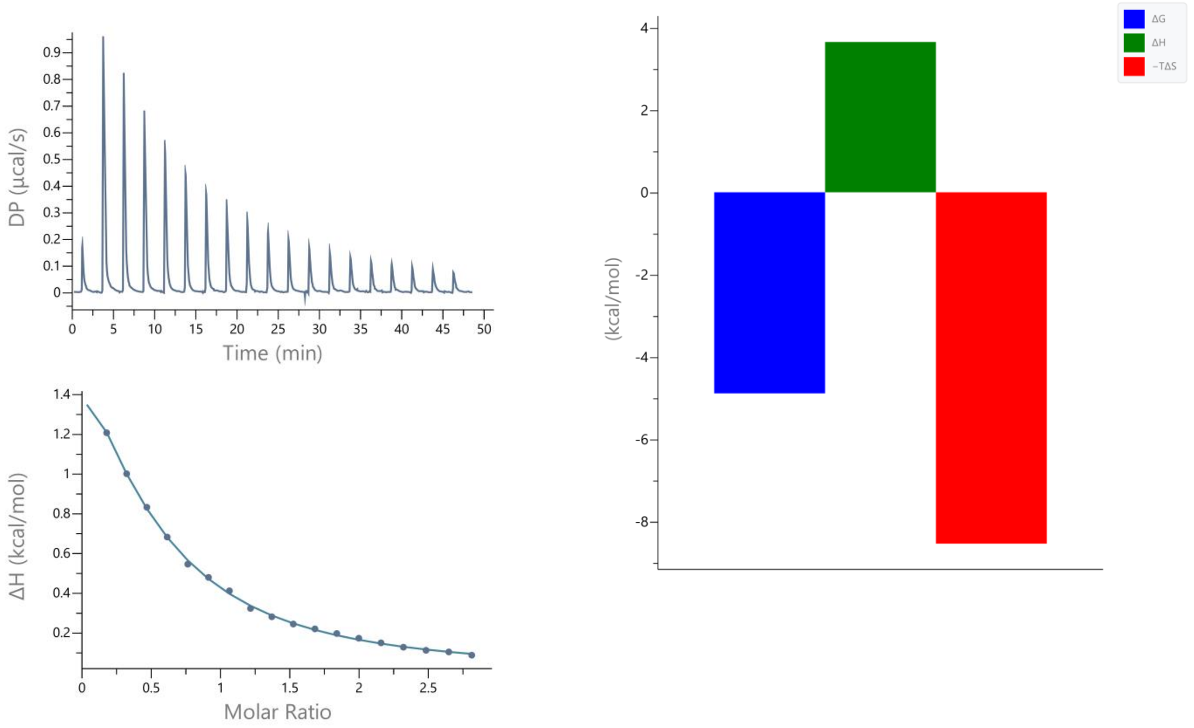

Figure S1. ITC results for titration of L1 with Zn(II) (left) and resulting Gibb's free energy graph.

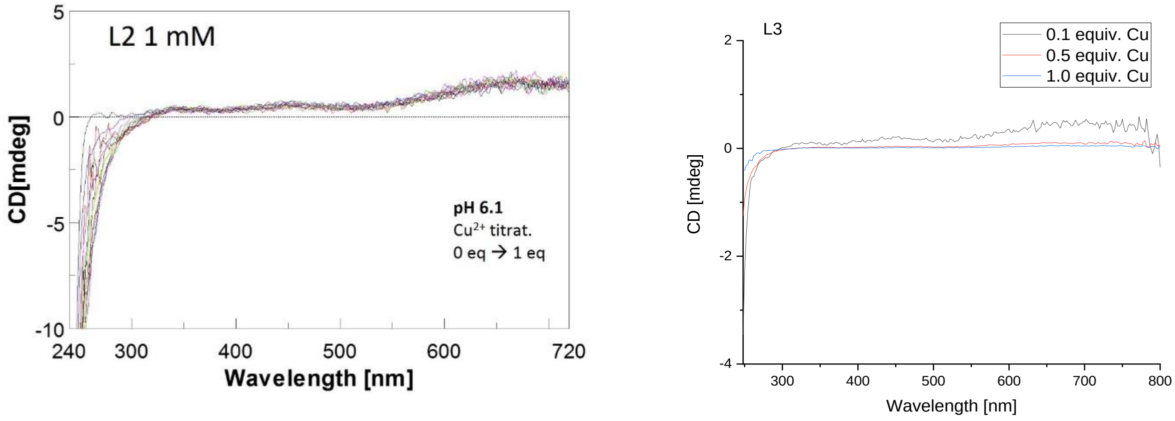

Figure S3. CD spectra for L2 (left) and L3 (right) titration with Cu(II) ions in HEPES.

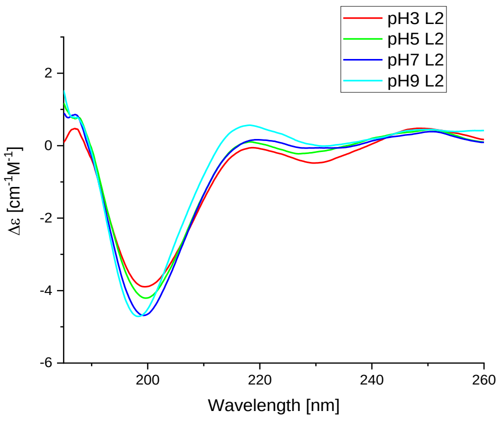

A

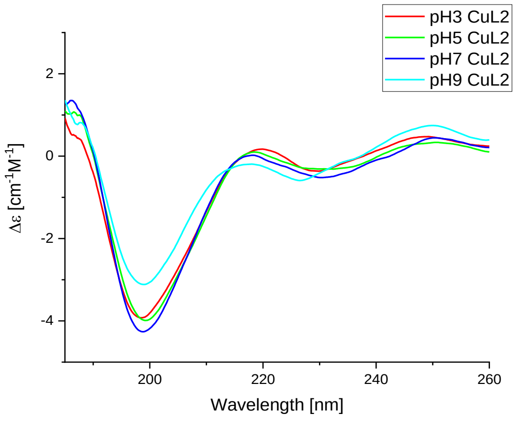

B

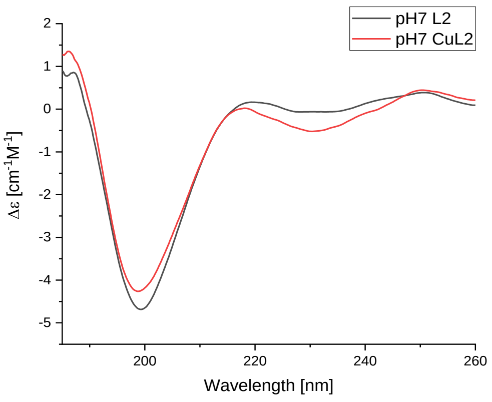

C

Figure S4. Far-UV CD results for L2-Cu(II) system; A) Titration of ligand; B) Titration of complex with Cu(II); C) Comparison of ligand and complex spectra in pH=7

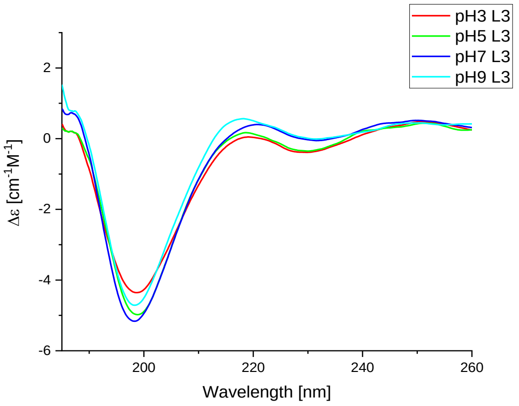

A

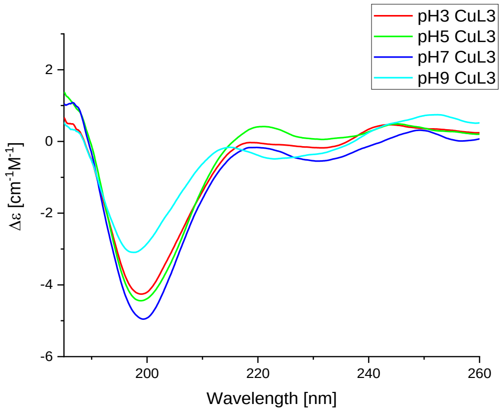

B

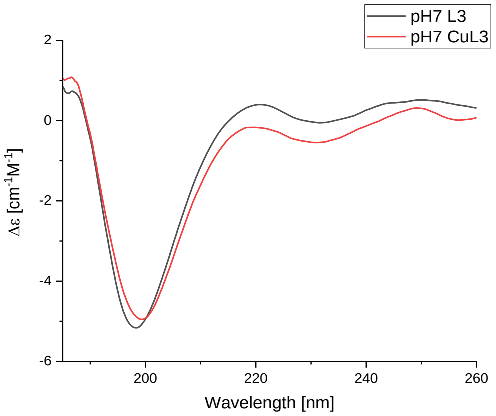

C

Figure S5. Far-UV CD results for L3-Cu(II) system; A) Titration of ligand; B) Titration of complex with Cu(II); C) Comparison of ligand and complex spectra in pH=7

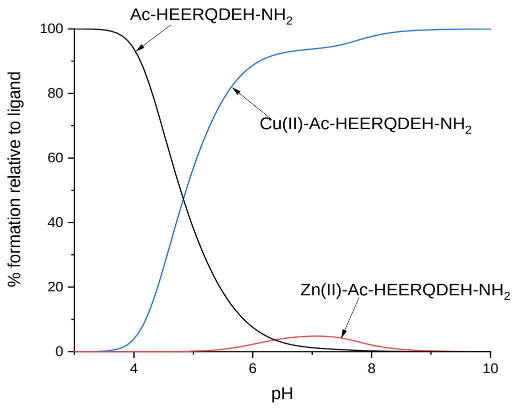

A

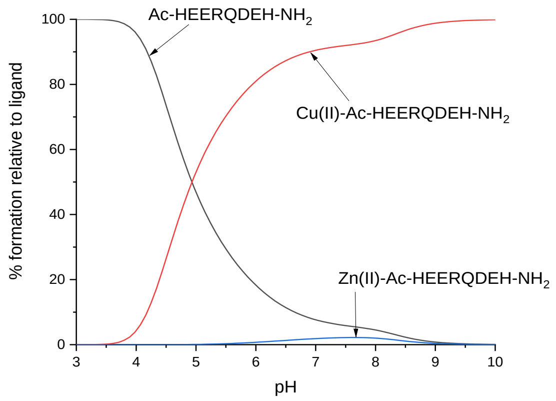

B

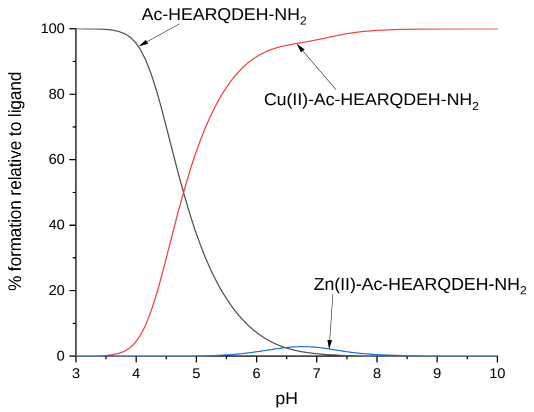

C

Figure S6. Competition plots for A) L1, B) L2, C) L4 in presence of Zn(II) ions. Previously calculated stability constants are applied to a theoretical situation, in which equimolar amounts of Zn(II) and both ligands are present, in 1:1:1 molar ratio.

References

[S1] L. D. Pettit, J. E. Gregor, H. Kozłowski, in R. W. Hay, J. R. Dilworth, K. B. Nolan (eds), *"Perspectives on Bioinorganic Chemistry"*, Vol 1, JAI Press, London, 1991, 1-41;

Supplementary Material should be uploaded separately on submission. Please include any supplementary data, figures and/or tables.

Supplementary material is not typeset so please ensure that all information is clearly presented, the appropriate caption is included in the file and not in the manuscript, and that the style conforms to the rest of the article.
